# The Aryl Hydrocarbon Receptor: A Mediator and Potential Therapeutic Target for Ocular and Non-Ocular Neurodegenerative Diseases

**DOI:** 10.3390/ijms21186777

**Published:** 2020-09-16

**Authors:** Mayur Choudhary, Goldis Malek

**Affiliations:** 1 Department of Ophthalmology, Duke University School of Medicine, 2351 Erwin Road, P.O. Box 3802, Durham, NC 27705, USA; 2 Department of Pathology, Duke University School of Medicine, Durham, NC 27705, USA

**Keywords:** aryl hydrocarbon receptor, inflammation, transcription factor, retinal diseases, neurodegeneration

## Abstract

The aryl hydrocarbon receptor (AHR) is a ligand-activated transcription factor, which senses environmental, dietary or metabolic signals to mount a transcriptional response, vital in health and disease. As environmental stimuli and metabolic products have been shown to impact the central nervous system (CNS), a burgeoning area of research has been on the role of the AHR in ocular and non-ocular neurodegenerative diseases. Herein, we summarize our current knowledge, of AHR-controlled cellular processes and their impact on regulating pathobiology of select ocular and neurodegenerative diseases. We catalogue animal models generated to study the role of the AHR in tissue homeostasis and disease pathogenesis. Finally, we discuss the potential of targeting the AHR pathway as a therapeutic strategy, in the context of the maladies of the eye and brain.

## 1. Introduction

Members of the animal kingdom, including humans, are subjected to an assortment of chemicals derived from their environment on a daily basis. Many of these chemicals may serve as signaling factors, relaying information about the environment, while others may be toxic, and as such must be processed and eliminated. Both vertebrate and invertebrate animals have evolved diverse mechanisms to detect, counter, and/or neutralize these chemicals.

Chemical surveillance may identify a broad range of exogenous chemical structures, through chemosensory receptors, which in turn trigger signaling pathways, resulting in a physiological response [1,2]. On a molecular level, animals take advantage of inducible enzymes and transporters to perform biochemical transformation and elimination of environmental toxic compounds and their metabolites. The enzymatic biotransformation of exogenous compounds is carried out by monooxygenases in the cytochrome P450 superfamily as well as conjugating enzymes such as glutathione transferases and glucuronosyl transferases [3,4,5]. The activity of these enzymes is augmented by transporter proteins, such as ATP-binding cassette (ABC) transporters, which act as efflux pumps to efficiently clear the metabolites of exogenous and xenobiotic chemicals from cells [6]. The final cog in the wheel is the sensory component, comprising members of the nuclear receptor superfamily as well as the aryl hydrocarbon receptor (AHR), a member of the basic helix-loop-helix-Per-ARNT-Sim superfamily of proteins [7,8,9,10].

Beyond performing xenobiotic metabolism, adjunct cellular functions have also been characterized for the AHR since its discovery in 1976 [11]. This is in part due to the assortment of AHR ligands that have been described, including the polychlorinated biphenyls, with the earliest one identified, 2,3,7,8-tetrachlorodibenzo-p-dioxin (TCDD) being the most widely studied one. TCDD has been found to display toxic potency in animals, closely associated with the induction of aryl hydrocarbon hydroxylase activity, known to be catalyzed primarily by cytochrome P450A1 (CYP1A1) [8,12,13]. The evolution of the CYP1A1-inducing function of the AHR coincides with its ability to bind halogenated and non-halogenated polycyclic aromatic hydrocarbons, signaling induction of its transcriptional activity [14]. Although originally believed to have a relatively narrow structural specificity, the AHR is now known to be somewhat promiscuous, and may bind to a wide range of chemical structures, including non-aromatic and non-halogenated compounds [15,16], as well as natural compounds derived from food. Some examples of endogenous ligands include flavonoids, indoles, indirubin, and indigo metabolites of arachidonic acid or the kynurenine pathway [16] (Table 1).

While the AHR has been shown to be involved in regulating biotransformation enzymes, it is clear from recent studies that it has more complex and diverse functions. Specifically, toxicities that occur following exposure to TCDD and related chemical compounds strongly indicate that the AHR, in addition to the induction of the CYP family of enzymes, is also involved in the regulation of physiological functions, such as immune responses, inflammation, neurodevelopment, thyroid and steroid hormone pathways, and reproductive function to name a few [40,41]. Cloning of the cDNA and gene in rodents has resulted in substantial progress in our understanding of the AHR pathway and its possible functions [42,43]. Significantly, characterization of the Ahr-null mice has revealed alterations in the liver, immune system, ovary, heart, eyes and other organs [44,45], further emphasizing the role of AHR-mediated regulation of numerous physiological processes, beyond toxin metabolism and clearance. In this review, we discuss studies which highlight the role of the AHR in select diseases of the central nervous system (CNS) and the functional contribution of the AHR in regulating the behavior of astrocytes, microglial and neuronal cells. Given that the retina is an extension of the brain, we also review new studies demonstrating the importance of the AHR in ocular biology and disease.

## 2. The AHR Signaling Pathway

**Figure 1:**
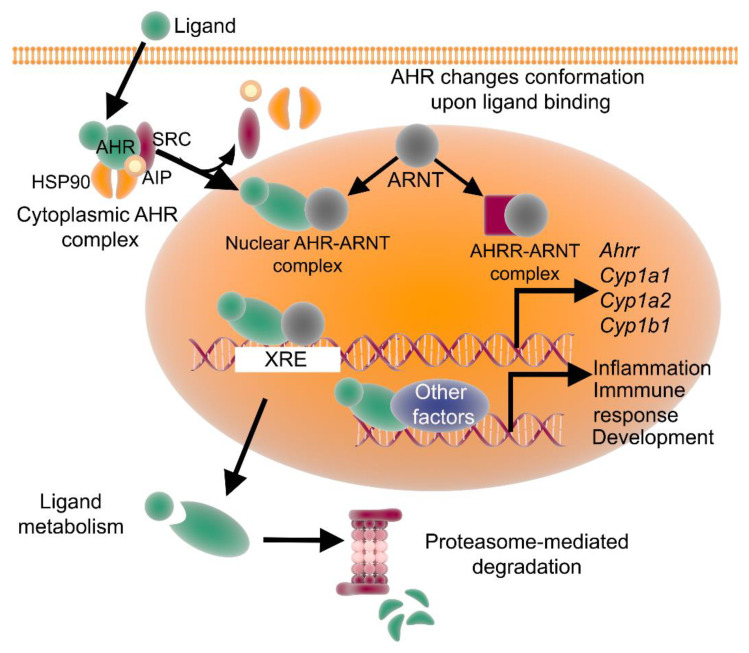
The AHR signaling pathway. In the classical pathway, an inactive form of the AHR is cytoplasmic and complexed with HSP90, AIP and SRC. Upon ligand binding, the AHR complex translocates to the nucleus, where the AHR forms a complex with ARNT and binds to XRE, inducing AHR-target gene expression. The AHR can also induce transcription of genes involved in inflammation, immune response and/or development. AHRR competes with the AHR for binding with ARNT and forms the inactive heterodimer AHRR-ARNT. The dissociation of the AHR transcriptional complex leads to translocation of the AHR to the cytoplasm, where it is degraded via the proteasomal pathway. AHR: aryl hydrocarbon receptor; AHRR: AHR repressor; ARNT: AHR nuclear translocator; AIP: AHR-interacting protein; Ub: ubiquitin; and XRE: xenobiotic response element.

The non-activated, non-ligand-bound form of the AHR is cytoplasmic and exists in a complex with several chaperones, including heat shock protein 90 (HSP90), a co-chaperone p23 and the hepatitis B virus X-associated protein 2 [46,47], which help maintain its secondary structure. AHR signaling mechanisms can be broadly classified into classical and non-classical pathways. In the classical pathway (Figure 1), ligand binding to the AHR results in a conformational change, leading to dissociation from the cytoplasmic complex and translocation into the nucleus, followed by binding to the aryl hydrocarbon receptor nuclear translocator (ARNT) to form an active heterodimer. The heterodimer then binds to the xenobiotic response element (XRE) and coregulator, modulating the expression of target genes [47]. One of the AHR target genes is the AHR repressor (*AHRR*), whose product competes with the AHR for binding to ARNT [48]. After the active heterodimer has performed its function, the AHR dissociates from ARNT, is exported out of the nucleus, covalently binds to ubiquitin, and is then degraded in the cytoplasm through the proteasomal pathway. This classical pathway has been validated by studies, which have shown that the level of the AHR protein can decrease rapidly following ligand-mediated activation, without an effect on the messenger RNA levels [49,50].

Non-classical pathways have also been reported in response to TCDD treatment including an increase in intracellular calcium concentration [51] and functional activation of the tyrosine kinase Src by releasing it from the AHR complex, followed by the activation of focal adhesion kinase [51,52]. Src activation has also been shown to be associated with rapid activation of the MAP kinase pathway. Physiologically and functionally, these non-classical pathways may converge leading to regulation of inflammation [53,54]. Interestingly, the glucocorticoid receptor pathway has been shown in a human retinal pigment epithelial cell line, to interact with the AHR pathway and modulate its transcriptional activity [55]. The AHR is also known to interact with transcriptional and epigenetic regulators to exert genome-wide effects [56,57], regulating histone acetylation and methylation, through species-specific and cell type-specific mechanisms [56,57,58]. AHR agonists can also cause the AHR–chaperone complex to induce signaling. For example, Src phosphorylates a number of target proteins including indoleamine 2,3-dioxygenase (IDO1) and members of the arachidonic acid and leukotriene pathways [53,59,60]. It should be noted that the AHR has also been suggested to act in a ligand-independent manner as observed in conditions of shear and oxidative stress, and studies with mutations in the AHR ligand-binding groove [61,62,63]. However, these results should be interpreted carefully, considering the inclusion of AHR ligands in the cell culture media, which can act as an additional means of AHR modulation [25,64].

Taking everything into account, the AHR pathway can exert its cellular and physiological effects through a number of mechanisms upon ligand binding. Though the AHR has been shown to be integral throughout the body, the remainder of this review will be devoted to its role in select neurodegenerative diseases and the potential of targeting it for therapeutic purposes. AHR nomenclature used in this review include AHR (human and mouse protein); *Ahr* (mouse gene); *AHR* (human gene); and Ahr (bacterial protein).

## 3. AHR Expression in the Central Nervous System (CNS)

Early studies on embryonic expression of Ahr and some of the components of the AHR pathway including *Arnt*, *Arnt2*, *Hif1a* and *Hif2a* in mouse tissue used in situ hybridization [65]. While Ahr mRNA was undetectable at embryonic age 9.5 days (E9.5d), the first signs of Ahr expression were observed at E13.5d in several tissues, most notably, in the primitive pituitary [65]. Ahr expression has also been observed in the neuroepithelium at E10–13d [66]. Notably, as development progressed, Ahr levels in the brain decreased, though Ahr expression levels have been found to be specific for cell type, organ/tissue, and developmental age, suggesting an important role in driving normal embryonic development. The expression of Ahr and its target genes have also been shown in astrocytes, microglia, and neurons of the cortex, cerebellum, and hippocampus of the CNS of pigs and rodents. Specifically, the brainstem and suprachiasmatic nucleus, which controls circadian rhythmicity, have shown markedly higher expression of Ahr than other regions of the brain [67,68]. AHR expression has also been detected in the spinal cord, as reported in the Human Protein Atlas [69]. The significance of Ahr in CNS glial and neuronal cells has been further illustrated by the phenotype of mice lacking functional AHR protein, impacting neurogenesis [70,71,72].

## 4. The AHR and Non-Ocular Neurodegenerative Diseases

Given the expression pattern of Ahr in the CNS, its role in neurodegenerative diseases has been an area of great interest. Ahr expression in neurons of normal rat brain has been found to be at low or moderate levels in the cortex and hippocampus, increasing following traumatic brain injury [73]. Interestingly, the localization of the AHR was mainly restricted to the cytoplasm, suggesting an inactivated state. This is important because the AHR has been shown to control neuronal cell cycle, differentiation and apoptosis, critical processes in the pathogenesis and recovery of brain injury [74,75]. In microglia, Ahr mRNA is upregulated as a result of inhibition of the microglia-specific activity of polycomb repressive complex 2, which leads to an aberrant acquisition of a clearance phenotype in striatal and cortical microglia, highlighting the link between the AHR and the clearance function of microglia [76]. The AHR has also been found to be activated and upregulated in lipopolysaccharide (LPS)-activated microglia, which can enhance the responsiveness of microglia to the AHR ligands. In the aforementioned study, the AHR activation caused by LPS alone induced proinflammatory and neurotoxic factors, whereas AHR activation by LPS along with 6-formylindolo(3,2-b)carbazole (FICZ) induced a microglial-mediated immune response, which was anti-inflammatory in nature [77], highlighting a potential link between AHR ligands and the microglial inflammatory response. In a model of experimental autoimmune encephalomyelitis (EAE), it has been observed that loss of the Ahr in astrocytes results in worsening of EAE [78], potentially due to upregulation in the expression of genes encoding chemokines (*Ccl2, Ccl20* and *Cxcl10*), cytokines (*Il6*, *Il12* and *Il23*) and pro-inflammatory markers (*Vim*, *Nos2* and *Csf2*). Since astrocytes can directly affect the activation state of microglia cells, the pro-inflammatory gene profile of Ahr-deficient astrocytes has the potential of exerting a neurotoxic effect in EAE. Ahr deletion in microglia also worsens EAE and increases demyelination and CNS monocyte recruitment. Additionally, Ahr loss results in upregulation of genes associated with microglial activation (*Apoe*, *Ddit4* and *B2m*), inflammation and neurodegeneration (*Ccl2*, *Nos2*, *Il1b* and *Il23a*) [79]. In astrocytes and microglia, the AHR acts by negatively regulating NF-κB activation, through suppression of cytokine signaling 2 [78,79].

An important aspect of the regulation of astrocyte/microglial function by the AHR is the role of metabolites generated by commensal flora and studies showing deficits in the levels of microbially-derived AHR agonists in conditions such as metabolic syndrome, multiple sclerosis (MS) and inflammatory bowel disease (IBD) [80]. It has been observed that the AHR agonists which are derived from the microbial metabolism of dietary tryptophan (Table 1) exert a therapeutic effect in inhibiting the functions of astrocytes and microglia, causing CNS inflammation and neurodegeneration. Microbial metabolite signaling mediated by microglial Ahr can also regulate the expression of TGFα and VEGFB, subsequently limiting the pro-inflammatory functions of the astrocytes [78,79,80]. These findings point to a gut–brain axis, modulated via the AHR signaling pathway.

The AHR has also been shown to mediate the expression of several genes encoding the protein components of the ubiquitin proteasome pathway, specifically, Ubch7, an E2 ubiquitin enzyme partner of parkin (PRKN) [81]. Parkin is an E3 ligase that catalyzes the ubiquitination of several proteins, including α-synuclein, synphilin-1, and Cdc-Rel, and plays a major role in the development of Parkinson’s disease (PD), the second most common neurodegenerative disease [82,83,84]. Mutations in the *PRKN* gene in humans are the most common cause of autosomal recessive early-onset parkinsonism and reduction on the levels of *PRKN* expression is linked to late-onset PD [85]. Additionally, *PRKN* haploinsufficiency contributes to a higher risk of sporadic PD [86]. Interestingly, PRKN is transcriptionally upregulated as a result of AHR activation [87]. Furthermore, the PRKN protein has been shown to be upregulated 3-fold in mouse ventral midbrain, and in silco analysis has revealed three AHR binding sites in the human *PRKN* promoter [87]. It has been observed that α-synuclein expression is reduced in the ventral midbrain upon AHR activation [87]. These observations point to a protective effect of AHR activation on the development of PD. Noteworthy is that several epidemiological studies have reported a negative correlation between PD and cigarette smoking, whose components include noted AHR agonists [88,89]. Similarly, tangeritin, a citrus flavonoid, AHR-activating ligand, is protective against parkinsonian effects induced in the 6-hydroxydopamine-treated rat model [90,91]. A different relationship has been reported between the AHR and another neurodegenerative disease, Alzheimer’s disease (AD). An increase in AHR expression in the astrocytes of aged individuals and AD patients has been observed [92], along with enhanced AHR protein levels in AD patients, while there were no differences between young and old donors. These studies suggest that the AHR is implicated in the aging process of the brain and possibly in the development of AD through its effects on astrocytes.

**Figure 2:**
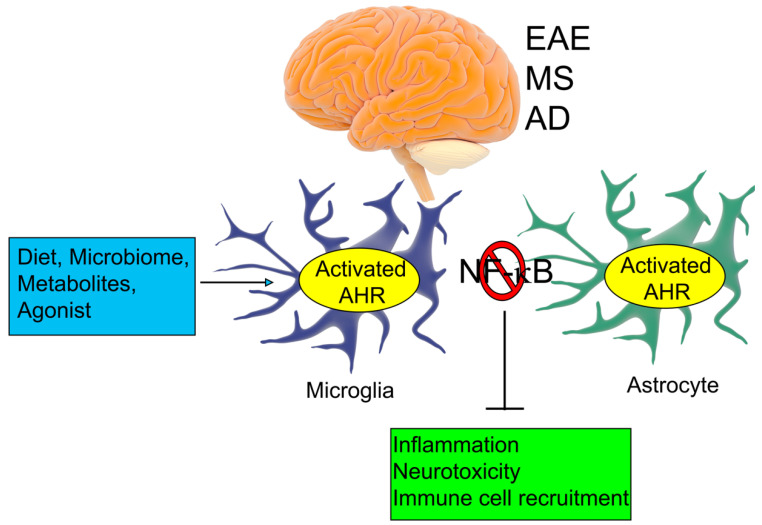
The AHR and neurodegeneration. AHR signaling is involved in the regulation of inflammation, neurotoxicity, and immune cell recruitment in various neurodegenerative diseases. The AHR regulates disease pathology via microglia and astrocytes in CNS. AHR agonists (endogenous and pharmacological) have been shown to inhibit NF-κB-mediated inflammatory signaling. EAE: experimental autoimmune encephalomyelitis; MS: multiple sclerosis; AD: Alzheimer’s disease.

With regards to harnessing the power of AHR activation for the treatment of CNS inflammation, laquinimod, a recently identified AHR agonist, has been shown to exert therapeutic immunomodulatory effects specifically in EAE and MS [93,94]. AHR activation by laquinimod not only leads to a decrease in axonal damage and demyelination, potentially by upregulating the expression of brain-derived neurotrophic factor (BDNF) [95], but also reduces infiltrating T cells and macrophages in the CNS [95]. Laquinimod has also been shown to have protective effects in MS by reducing brain atrophy and inhibiting astrocyte activation and neurodegeneration [93,96]. These studies support pursuing the AHR as a target to develop and test potential therapies for the management of CNS diseases, characterized by a pro-inflammatory phenotype of astrocytes and microglia (Figure 2).

## 5. AHR Expression in the Eye

The retina is an extension of the brain and an important part of the nervous system. Though the role of the AHR in the nervous system has been under investigation for some time, studies reporting on the expression of AHR in human and mouse ocular tissues are scarce in number. In human fetal tissue, AHR staining has been observed in the retina [97], while no staining has been seen in the choroid and scleral tissue. RNA-seq profiling of purified rod photoreceptors, retinal cells that function primarily in low-light conditions, showed an increase in Ahr from P2 to 18 months of age, suggesting a critical role of Ahr in rod photoreceptors through the greater part of the lifespan of mice [98]. Additionally, retinal gene expression profiling data in mice has revealed an increase in Ahr gene expression from P0 to P21 [98]. In adult human ocular tissue, the expression of AHR has been reported to also be present in human retinal pigment epithelial cells (RPE), nurse cells supporting the neural retina and forming the outer blood-retina barrier [99]. This report constructed a nuclear receptor atlas of 48 members of the nuclear receptor superfamily as well as AHR and *ARNT*, from RNA isolated from freshly isolated human RPE cells, human primary RPE cell lines and the spontaneous human RPE cell line, ARPE19. The age of the donors for the freshly isolated RPE and primary cell lines ranged from 51 to 76 years old. Remarkably, AHR mRNA levels were classified in the highly expressed category in RNA isolated from all three sources [99]. Additional reports have shown that AHR and its obligate binding partner *ARNT* are expressed in ARPE19 cells, primary human RPE cells, a choroidal endothelial cell line called RF/6A, and freshly isolated retina, RPE and choroid from human donor eyes [100,101]. At a functional level, baseline transcriptional activity has been found to be negatively correlated with age in primary RPE cells, with RPE cell lines established from older donors (58–67 years old) showing a significant reduction in AHR activity as compared to RPE cell lines established from young donors (7–51 years old). Intriguingly, the mRNA levels did not show a significant difference between the two cohorts, but protein levels showed a trend towards decline in the older cohort [100]. Microarray analyses of human donor tissue (aged 49–76 years old), specifically, iris epithelium and RPE, have shown that older iris epithelial cells and relatively young RPE cells show higher AHR expression, leading to the hypothesis that older iris epithelial cells maintain AHR-related detoxification capacities during their lifespan, whereas the RPE cells do not, supporting the findings by Hu et al. [100,102]. In summary, AHR/Ahr expression studies performed in human and mouse ocular tissue highlight the critical role of this receptor during embryonic development, throughout life, and in the course of aging, in ocular tissue.

## 6. The AHR and Ocular Diseases

The AHR has been shown to be associated with various maladies which impact the eye. It has been shown that a homozygous loss-of-function mutation in the AHR gene is associated with autosomal foveal hyperplasia with infantile nystagmus, which is recessively inherited [103]. Comparably, constitutive loss of Ahr (Ahr*^−/−^)* in adult mice leads to a similar phenotype, characterized by abnormal eye movements in the form of a spontaneous pendular horizontal nystagmus [104]. These mice also exhibit a decline in their optokinetic responses, possibly due to deficits in the visual or visuomotor circuitry. The role of the AHR in physiological processes and development of the CNS is further highlighted by the faulty control of ocular movement, a drop in optic nerve myelin sheath coverage and presence of subretinal microglial cells [98,101,105]. In a follow-up study, it was shown that that infantile nystagmus in Ahr*^−/−^* mice coincides with defects in early processing of visual information, as indicated by lower amplitudes of visual evoked potentials (VEPs) from the primary visual cortex [105]. Furthermore, these mice presented with an altered optic nerve myelin sheath. Collectively, these studies provide evidence in support of the proposed role of the AHR in maintaining myelination of the visual and optomotor pathway.

The AHR has also been proposed to play a potential role in the susceptibility of age-related macular degeneration (AMD), the most common cause of visual impairment in individuals over 65 years old [100,101,106]. Since AHR expression was detected at high levels in RPE cells, a cell type severely impacted in the progression of AMD, the potential role of the AHR in AMD pathogenesis has been investigated with focus on RPE cells [99]. Further support for studying the AHR in AMD comes from the fact that the AHR regulates numerous molecular pathways, which overlap with purported AMD pathogenic pathways, including extracellular matrix proteolysis, cellular degradation, and clearance. Hu et al. showed that though AHR is expressed in human RPE cells, the activity of the receptor decreases with age. Furthermore, aged Ahr*^−/−^* mice, generated by targeted deletion of exon 2 of Ahr on the C57BL/6J background, exhibit some hallmark features of the dry clinical subtype of AMD, prevalent in approximately 85% of AMD patients. These clinical features include a decline in visual function, formation of lipid-rich sub-RPE deposits, increased RPE autofluorescence and RPE atrophy [100,107]. Furthermore, these mice display choroidal thinning, suggesting Ahr regulation of the integrity of the choroidal vasculature. Though spontaneous choroidal neovascularization (CNV), a characteristic feature of the neovascular or wet clinical subtype of AMD, has not been observed in aged Ahr*^−/−^* mice, they are more susceptible to severe choroidal neovascular lesion formation in a laser-induced injury model [101]. The AHR in the eye has been shown to be involved in the regulation of multiple pathways critical in AMD, namely, inflammation, angiogenesis and extracellular matrix regulation in the outer retina. A higher influx of immune cells and deposition of extracellular matrix components, including collagen type 4 and fibronectin, in CNV lesions, has also been observed in aged mice. Notably, AHR activation by leflunomide and flutamide ameliorates the severity of the choroidal neovascular lesions demonstrating potential therapeutic effects, which appear to be mediated by a reduction in angiogenesis and fibrosis [108]. The ocular phenotype of Ahr*^−/−^* mice, generated by targeted deletion of exon 1 on C57BL/6N background also reveals retinal changes including accumulation of autofluorescent subretinal microglial cells, RPE atrophy and immune activation [98]. Most recently, a synthetic AHR ligand 2,2′-aminophenyl indole (2AI) has been reported to act as a strong agonist and shown to protect RPE cells from cytotoxicity induced by lipid peroxidation products including 4-hydroxynonenal, potentially by increasing the intracellular levels of palmitoleic acid [109]. Further, 2AI was also shown to have a therapeutic effect in ameliorating light-induced retinal degeneration in mice [109]. The AHR pathway also contributes to choroidal γδ T cell-mediated protection of RPE cells and retina [110]. These findings support a protective role of the AHR in toxin and lipid clearance, and a wide-ranging impact of AHR loss in the pathology and etiology of AMD.

Genetic studies have revealed an association between AHR and retinitis pigmentosa (RP), a group of hereditary degenerative diseases affecting the retina. Clinical manifestations of RP include night blindness, progressive loss of peripheral vision in the early, and complete vision loss in the late, stages of the disease [111]. Zhou et al. identified a homozygous splicing mutation (c.1160+1G > A) in the AHR gene in an autosomal recessive RP family from India by whole-exome sequencing [112]. This mutation leads to skipping of exon 9 and loss of AHR transcriptional activity. This observation was further corroborated by generating a retina-specific Ahr knockout mouse model, which exhibited late-onset retinal degeneration, a decline in visual function, a reduction in photoreceptor cells, and an increase in apoptotic cell death in the retina, suggesting that Ahr is a candidate gene for the pathogenesis of RP [112]. A congenital form of RP, called Leber Congenital Amaurosis (LCA), is also associated with the AHR, indirectly through aryl hydrocarbon interacting protein-like 1 protein (AIPL1) [113]. LCA is a clinically and genetically heterogenous disease with severe vision loss from birth. It is usually inherited as an autosomal recessive trait and characterized by chorio-retinal atrophy, macular aplasia, intraretinal pigment migration, and constricted retinal blood vessels. Functionally, rod and cone responses are severely diminished [114,115]. The AIPL-related LCA exhibits a severe phenotype characterized by visual impairment, maculopathy, optic disc pallor and a considerable prevalence of cataracts [113]. Loss-of-function mutations in AIPL lead to a decline in photoreceptor function, as AIPL is expressed in rod and cone cells during development as well as in mature rod photoreceptors [116,117]. 

The AHR pathway has also been linked, indirectly, to the pathogenesis of primary open-angle glaucoma (POAG) through its target gene *CYP1A1*. Glaucoma is a genetically heterogenous optic neuropathy, characterized by the loss of retinal ganglion cells, leading to optic nerve damage and subsequent vision loss [118]. Intraocular pressure is an important risk factor in POAG. Two polymorphisms in the *CYP1A1* gene have been shown to be associated with POAG, namely the CYP1A1m1 (T to C transition in exon 7) and CYP1A1m2 (Valine to Isoleucine transition at codon 462) [119]. Additionally, the association of *CYP1B1* has also been shown in primary congenital glaucoma, which manifests itself with birth or at infancy [120]. Notably, CYP1A1m1 allele is located in a region near loci that have been linked to glaucoma, namely *GLC1I* [121]. These genetic studies underscore the role of the AHR in regulating the pathogenesis of multiple ocular disorders.

**Figure 3:**
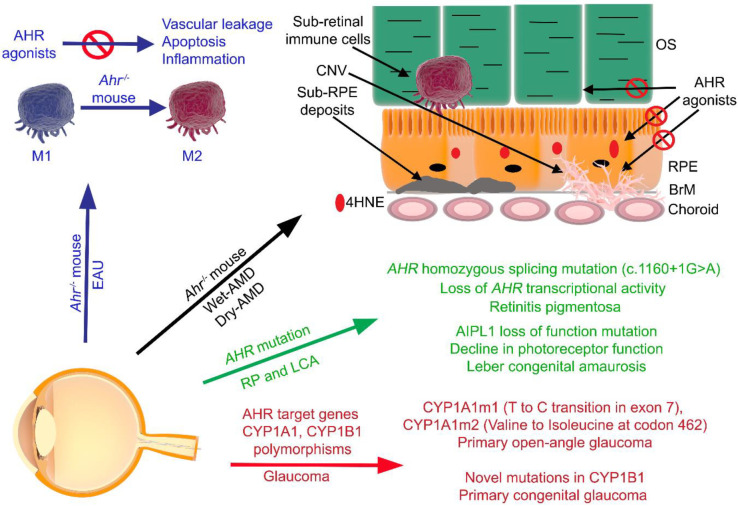
The AHR and ocular pathologies. AHR signaling is involved in the regulation of numerous pathogenic pathways in ocular diseases, such as the dry and wet clinical subtypes of age-related macular degeneration (AMD) and uveitis. Much of the ocular pathologies known to be regulated by the AHR were discovered by careful characterization of the ocular phenotype of Ahr*^−/−^* mice. Notable pathogenic pathways in AMD include inflammation, angiogenesis, and lipid metabolism. AHR agonists have been shown to inhibit vascular leakage, apoptosis, inflammation and accumulation of sub-retinal immune cells. Treatment with AHR agonists has been shown to have a therapeutic effect in protecting RPE cells from lipid peroxidation-induced toxicities, choroidal neovascularization, and uveitis. Primary open-angle glaucoma and primary congenital glaucoma have been shown to be associated with polymorphisms in AHR target genes cytochrome p450 A1 and B1 (CYP1A1 and CYP1B1). Retinitis pigmentosa (RP) and Leber congenital amaurosis (LCA) have been shown to be associated with mutations in AHR as well as *AIPL1*. AIPL1: Aryl-hydrocarbon-interacting protein-like 1; 4HNE: 4-hydroxynonenal; CNV: choroidal neovascularization; AMD: age-related macular degeneration; OS: photoreceptor outer segments; RPE: retinal pigment epithelium; BrM: Bruch’s membrane.

Exposure to AHR-activating toxicants has been reported to contribute to the development of immune disorders and given that the AHR plays a critical role in regulating immune responses, it is important to discuss the interactions between the AHR pathway and the pathogenesis of inflammatory disorders with ocular indications [54,122,123]. *AHR* message levels have been found to be reduced in peripheral blood mononuclear cells and macrophages isolated from patients of Behcet’s disease, which is a chronic systemic inflammatory disease affecting the eye, skin, oral, mucosa, gastrointestinal tract and central nervous system [124,125,126]. The AHR has also been shown to regulate inflammation via the STAT3–SOCS3 pathway, leading to retinopathy in Ahr*^−/−^* mice, as a result of carcinogen, benzo(a)pyrene treatment. In vitro treatment performed in a human RPE cell line has revealed that the AHR upregulates SOCS3 expression in response to benzo[a]pyrene treatment leading to protection of RPE cells against inflammatory damage [127]. Choroidal γδ T cells secrete various cytokines which are involved in the maintenance of RPE cell integrity and support the recovery of the overlying retina in response to injury. Incidentally, γδ T cells isolated from Ahr*^−/−^* mice lack the immunosuppressive and restorative properties of γδ T cells, suggesting a functional role of the AHR pathway [110]. Treatment with AHR agonists, 6-formylindolo(3,2-b)carbazole (FICZ) and 2-(1’H-indole-3’carbonyl)-thiazole-4-carboxylic acid methyl ester (ITE), can inhibit interferon-gamma (IFN-*γ)*, interleukn-17 (IL-17), and induced IL-22 production by PBMCs, leading to a decrease in overall inflammation. Similarly, AHR activation has shown therapeutic effects in an experimental model of uveitis, which is an intraocular inflammatory disease [128,129]. It has also been shown that the AHR is involved in regulating apoptosis and inflammation in experimental autoimmune uveitis (EAU). Ahr*^−/−^* mice subjected to EAU show evidence of significantly increased macrophage presence and stronger polarization from the M2 (involved in tissue repair) to the M1 (proinflammatory) phenotype compared to Ahr*^+/+^* mice [129]. Ahr*^−/−^* EAU mice also display higher protein expression levels of the M1 marker subset, inducible nitric oxide synthase (iNOS) and CD16, and lower protein expression levels of the M2 marker subset, arginase-1 (Arg-1) and CD206, relative to Ahr*^+/+^* EAU mice [129]. Finally, AHR agonists ITE and FICZ have been shown to prevent TGFβ-induced myofibroblast formation in fibroblasts, specifically via inhibiting the β-Catenin/Wnt signaling, leading to the regulation of contractility and proliferation of the human orbital fibroblasts isolated from patients with thyroid eye disease [130]. All in all, though investigating the AHR in the eye is a relatively new area of research, the studies so far indicate that this receptor is involved in modulation of pathogenic pathways, which regulate several retinal diseases (Figure 3) and should be considered as a potential druggable target for the treatment of such maladies.

## 7. Concluding Remarks and the Future of AHR-Targeted Therapies for Ocular Diseases

The AHR is an evolutionally conserved transcription factor that assimilates environmental, metabolic and endogenous inputs into precisely targeted cellular responses, which are vital to pathogenic processes in ocular and non-ocular neurodegenerative diseases (Table 2). However, the response of the AHR pathway is complex, and displays cell type, ligand, and tissue specificity. The various facets of AHR activation, namely, classical and non-classical pathways, add another layer of complexity to the overall process. This confounds the extrapolation of in vitro results to animal disease models and warrants a greater degree of optimization and study. Therefore, it is imperative that future studies focus on specific aspects of the pathway, including ligand type, cellular and tissue specificity, in vitro versus in vivo complexity, acute versus chronic modulation, and local versus systemic delivery in order to maximize the potential of targeting the AHR for therapeutic purposes of ocular and non-ocular neurodegenerative diseases.

## Figures and Tables

**Table 1:** List of select endogenous ligands of the AHR pathway.

	Ligand	Biochemical Pathway	Ref
Dietary compounds	Indole-3-acetonitrile (I3ACN)	Dietary/Cruciferous vegetables	[17]
	Indole-3-carbinole (I3C)	Dietary/Cruciferous vegetables	[17,18,19]
	3,3′-diindolylmethane (DIM)	Dietary/Cruciferous vegetables	[17,20]
	Indolo (3,4) bicarbazole (ICZ)	Dietary/Cruciferous vegetables	[17,21]
Tryptophan metabolites	L-Kynurenine (Kyn)	IDO1, IDO2 and TDO	[22,23]
	Kynurenic acid (KA)	IDO1 and IDO2	[24]
	6-Formyl indolo (3,2-b) carbazole (FICZ)	UVB-dependent pathway	[25,26]
	Indoxyl-3-sulfate (I3S)	Microbial and host metabolism	[27]
	Indirubin	Microbial and host metabolism	[28,29]
	Tryptamine	Microbial metabolism	[30,31]
	3-Methylindole	Microbial and host metabolism	[32,33]
	2-(1′H-indole-3′-carbonyl) thiazole-4-carboxylic acid methyl ester (ITE)	Tryptophan and cysteine metabolism	[34,35]
Other metabolites	Bilirubin	Heme metabolism	[36,37]
	Biliverdin	Heme metabolism	[38]
	12(R)-hydroxy-5(Z),8(Z),10(E), 14(Z)-eicosatetraenoic acid (12R-HETE)	Arachidonic acid metabolite	[39]

**Table 2:** The role of the AHR in retinal and neurodegenerative diseases.

Disease Model	AHR Status	Therapeutic AHR Activation	Ref
Infantile nystagmus (Human)	c.1861C>T; p.Q621 *	Not tested	[103]
Nystagmus	Ahr*^−/−^* (exon 2) C57BL/6J	Not tested	[104]
Defect in VEPs	Ahr*^−/−^* (exon 2) C57BL/6J	Not tested	[104]
AMD (dry)	Ahr*^−/−^* (exon 2) C57BL/6J	Not tested	[100]
	Ahr*^−/−^* (exon 1) C57BL/6N		[98]
AMD (neovascular)	Ahr*^−/−^* (exon 2) C57BL/6J	Tested Leflunomide/Flutamide	[108]
Behchet’s disease (Human)	Low AHR in macrophages	Tested, FICZ, ITE	[125,126]
Uveitis	AHR activation	Tested, ITE	[35,129]
EAE	AHR activation in microglia	Tested, LPS and FICZ	[77]
	Ahr loss in astrocytes	Tested, Trp metabolites, I3S	[78,79]
EAE and MS		Tested, Laquinimod,	[93,94,95,96]
PD		Tested, Tangeretin	[87,91]
AD	High AHR in hippocampus and astrocytes	Not tested	[92]

* is used for 3’-UTR. AD: Alzheimer’s disease; AMD: age-related macular degeneration; EAE: experimental autoimmune encephalomyelitis; FICZ: 6-formyl indolo (3,2-b) carbazole; I3S: indoxyl-3-sulfate; ITE: 2-(1’H-indole-3’carbonyl)-thiazole-4-carboxylic acid methyl ester; LPS: lipopolysaccharide; MS: multiple sclerosis; PD: Parkinson’s disease; Trp: tryptophan metabolites; VEP: visual evoked potential.
